# Advanced glycation end product (AGE) modified proteins in tears of diabetic patients

**Published:** 2010-08-11

**Authors:** Zhenjun Zhao, Jingfang Liu, Bingyin Shi, Shuixiang He, Xiaoli Yao, Mark D.P. Willcox

**Affiliations:** 1 Brien Holden Vision Institute, Sydney, Australia; 2 First Hospital Affiliated to Medical College, Xi’an Jiaotong University, Xi’an, China; 3 The School of Optometry and Vision Science, University of New South Wales, Sydney, Australia

## Abstract

**Purpose:**

High glucose level in diabetic patients may lead to advanced glycation end product (AGE) modified proteins. This study investigated AGE modified proteins in tears and compared their levels in diabetic patients (DM) with non-diabetic controls (CTL).

**Methods:**

Basal tears were collected from DM with (DR) or without (DNR) retinopathy and CTL. Total AGE modified proteins were detected quantitatively by a dot immunobinding assay. The AGE modified proteins were separated in 1D- and 2D-SDS gels and detected by western-blotting. The individual AGE modified proteins were also compared between groups using densitometry.

**Results:**

Compared with the CTL group, tear concentrations of AGE modified proteins were significantly elevated in DR and DNR groups. The concentration of AGE modified proteins in diabetic tears were positively correlated with AGE modified hemoglobin (HbA1c) and postprandial blood glucose level (PBG). Western blotting of AGE modified proteins from 1D-SDS gels showed several bands, the major one at around 60 kDa. The intensities of AGE modified protein bands were higher in DM tears than in CTL tears. Western blotting from 2D-SDS gels showed a strongly stained horizontal strip, which corresponded to the major band in 1D-SDS gels. Most of the other AGE modified protein species were within molecular weight of 30–60 kDa, PI 5.2–7.0. Densitometry analysis demonstrated several AGE modified proteins were elevated in DR or DNR tears.

**Conclusions:**

Total and some individual AGE modified proteins were elevated in DM tears. AGE modified proteins in tears may be used as biomarkers to diagnose diabetes and/or diabetic retinopathy.

## Introduction

The human tear fluid has a complex multilayered film structure [1]. Although up to six layers have been proposed, most researchers agree that some form of three-layered structure is probably operational in the normal eye, comprising an extensive aqueous layer situated between a mucin layer and a lipid layer [2,3]. Maintenance of this structure is important in performing tear film function and minimizing tear fluid evaporation [4,5]. The aqueous layer, which is mainly secreted from the lacrimal gland, contains locally synthesized and other sources, such as serum, derived proteins [6,7]. Quantitatively, the major tear proteins are lysozyme, tear lipocalin, secretory immunoglobulin A (sIgA), and lactoferrin [8]. Nearly 500 other less abundant proteins have also been reported in human tear fluid [9-11].

One of the fundamental functions of human tear film is to protect the cornea and conjunctiva and keep them healthy. Protein components are vital in achieving this goal [12]. To help prevent corneal or conjunctival infection, tear proteins such as lactoferrin, lysozyme and complement proteins comprise the non-adaptive antimicrobial factors, and sIgA comprises the predominant adaptive protein [13]. Other tear proteins such as tear lipocalin, a lipid-binding protein in tears, may help stabilize the tear film [14,15]. Tear proteins have other functions such as promoting wound healing [16] by affecting the migration of ocular surface epithelial cells. Bioactive proteins and peptides such as cytokines have also been detected in tears and they may participate in regulating the biochemical processes inside corneal and conjunctival epithelial cells [10,17].

Studies have demonstrated that glucose exists in tears and its level is elevated in diabetic (DM) patients [18,19]. In theory, tear proteins, especially those derived from blood or ocular surface epithelial cells with a long half-life in the body, could undergo glycation (non-enzymatic glycosylation) in this pathological condition [20] due to a spontaneous sugar concentration-dependent chemical (non enzymatic) reaction of reducing sugars with the amino groups of proteins. Glycation results in the production of advanced glycation end product (AGE) modified proteins which might impair protein function. In addition, AGE modified proteins have been reported to participate in many DM complications [21,22], including diabetic retinopathy (DR) [23,24]. Increased level of these proteins in tears may be an indicator of retina damage and also may affect the ocular surface. To the best of our knowledge, there is no report regarding AGE modified proteins in tears, although AGE modified proteins in blood [25,26] and other organs [27-32] have been the subject of many previous studies, and AGE modified hemoglobin (HbA1c) has been used to monitor DM for many years [33].

The eye is very sensitive to high blood glucose levels. DR, the most frequent diabetic microvascular disease, is the most frequent cause of new cases of blindness among adults aged 20–74 years [34]. During the first two decades of disease, nearly all patients with type 1 DM and >60% of patients with type 2 DM develop DR [34]. The ocular surface can be affected by DM and reduced corneal sensitivity [35] and altered tear quantity and quality are observed in DM patients [36]. Dry eye syndrome, an ocular surface disease, is frequently found in DM patients, and a positive correlation was found between HbA1c level and the presence of dry eye syndrome [37].

As the first step to elucidate the effect of AGE modified proteins on diabetic eye complications, we analyzed AGE modified proteins in tears from DM patients with or without retinopathy.

## Methods

### Study subjects

All subjects were recruited from the Department of Endocrinology, First Hospital Affiliated, Medical School of Xi’an Jiaotong University, Xi'an, China. DM was diagnosed according to the 1999 World Health Organization criteria. DR, including non-proliferative DR (NPDR) and proliferative DR (PDR), was defined on the basis of fundus examination and fluorescein angiography. NPDR is characterized by microaneurysm, hemorrhage, exudate, macular ischemia, macular edema on the retina, and PDR characterized by abnormal new vessel formation on the retina, vitreous hemorrhage, and traction retinal detachment.

Forty-eight type 2 DM patients without retinopathy (DNR), 49 patients with retinopathy (DR) and 50 sex- and age-matched non-DM controls (CTL) were recruited. Due to the small amount of tears that can be collected, samples in each group were randomly separated into three subgroups, i.e., tears for dot-immunobinding assay, tears for one-dimensional (1D) gel analysis, and tears for two-dimensional (2D) gel analysis, respectively (see below, Table 1). Test results of fasting blood glucose (FBG), postprandial blood glucose (PBG), and HbA1c levels, measured by standard procedures used in pathology laboratories, were collected for all DM patients. Patients’ age, gender and duration of diabetes were recorded. Student’s *t*-test showed that there were no significant differences between DNR and DR groups for any of the parameters except for diabetic duration in the 2D gel analysis subgroup (Table 1).

**Table 1:** Clinical characteristics of the diabetic patients and non-diabetic controls.

**Group**	**Age (years)**	**Sex (M/F)**	**Diabetes duration (years)**	**FBG (mmol/l)**	**PBG (mmol/l)**	**HbA1c (%)**
**Samples used in dot immunobinding assay**						
CTL	59.0±9.4	8/7	-	-	-	-
DR	61.3±7.6	7/8	8.0±6.8	9.1±2.4	12.4±3.7	10.0±2.4
DNR	61.1±8.4	8/7	9.0±4.5	7.7±3.4	11.8±4.2	9.9±2.6
**Samples used in 1D gel analysis**						
CTL	54.4±12.0	3/2	-	-	-	-
DR	64.5±5.0	2/2	9.8±9.4	8.2±1.5	10.4±1.7	8.0±2.6
DNR	68.5±5.0	1/2	8.5±0.7	7.9±1.0	10.4±1.0	7.0±0.9
**Samples used in 2D gel analysis**						
CTL	57.3±8.6	15/15	-	-	-	-
DR	59.1±10.6	15/15	11.0±6.1	9.4±2.8	14.1±5.5	10.0±2.3
DNR	57.3±11.2	15/15	5.9±4.6*	9.7±3.7	15.6±6.8	9.8±2.8

In the table, CTL: non-diabetic controls; DR: type 2 diabetics with retinopathy; DNR: type 2 diabetics without retinopathy; FBG: fast blood glucose; PBG: postprandial blood glucose; HbA1c: glycosylated hemoglobin. Data were expressed as mean±standard deviation. The asterisk indicates a p<0.01.

Before enrolment in the study, all subjects signed an informed consent after explanation of the nature and possible consequences of the study. All experimental protocols were reviewed and approved by the hospital Human Ethics Review Committee and complied with the Declaration of Helsinki for Experimentation on Humans, 1975 and revised in 1983.

### Tear samples

Open eye basal tear samples were collected by a blunt glass capillary tube to obtain 5–10 µl tears from the outer canthus of the eye. Total protein concentration of each sample was assayed using a Lavapep protein quantification kit (Fluorotechnics, Gladesville, NSW, Australia) according to the manufacturer’s instruction. The tear samples were stored at −80 °C until tested.

### Dot-immunobinding assay of AGE modified proteins

Aliquots of the tear samples were diluted 1:20 in PBS (pH 7.4). Four µl of the diluted tear samples and various concentrations of glycated BSA (AGE-BSA; Sapphire Bioscience, Redfern, NSW, Australia) were dotted on nitrocellulose (NC) membrane at 1 cm intervals and allowed to dry for 1 h at ambient temperature (AT). Unreacted protein-binding sites on the membrane were blocked by immersing the membranes in 3% BSA in Tris-buffer saline (TBS, 10 mM Tris base and 150 mM NaCl, pH 7.5) and incubated for 2 h at AT, followed by washing three times with 0.05% Tween-20 in TBS (TBST). NC membranes were incubated with rabbit anti-human AGE polyclonal antibody (Sapphire Bioscience, Redfern, NSW, Australia) diluted 1:1,000 in blocking solution containing 0.5% BSA (BMBA) for 2 h at AT. After washing three times with TBST, the membrane was incubated with secondary antibody (goat anti-rabbit IgG peroxidase-labeled; Bio-Rad, Hercules, CA) diluted 1:10,000 in BMBA for 1 h at AT. Luminal/enhance and peroxidase buffer solutions (Immun Star WesternC Kit 170–5070; Bio-Rad) in a 1:1 ratio were added to the membrane after another three washes and incubated for 3–5 min. The chemiluminescent spots were detected using a Versa Doc Imaging System (Bio-Rad). Quantity-one software (Bio-Rad) was used to analyze the image. Standard curves were generated using the AGE-BSA standards (1,000, 500, 250, 125, 62.5, 31.2 and 15.6 ng/ml) and were used to calculate the concentrations of AGE modified proteins in tear samples. The results were also divided by total protein concentration to convert to amount (µg) of AGE modified proteins per mg of total tear proteins.

The antigen to which this polyclonal-AGE antibody was raised was a proprietary mixture of AGE-human serum albumin and AGE-BSA (Abcam, product datasheet). The antibody reacts with several different AGE moieties, such as N^ε^-(carboxymethyl)lysine, imidazolone and others, but has minimal reactivity (<1%) to purified HSA and BSA (product datasheet).

### Western-blotting detection of AGE modified proteins in 1D-SDS gels

Three µl tear samples (20–30 μg total protein) were mixed with 1 µl of 4× sample buffer (0.125 M Tris-HCl, 2% SDS, 40% v/v glycerol, 0.8% bromophenol blue, pH 6.8). Following incubation at AT for 10 min, samples were loaded onto a pre-cast Bio-Rad 4%–12% Bis-Tris 1.0 mm minigel. Electrophoresis was performed at 100 V in running buffer (25 mM Tris base, 0.1% SDS, 192 mM glycine, pH 8.3) until the dye front reached the end of the gel. After soaking the gel in equilibrating buffer (25 mM Tris base, 192 mM glycine, 20% methanol, pH 8.3) for 30 min, the proteins were electro-transferred to NC membrane using a Bio-Rad mini**-**gel transfer apparatus in transfer buffer (250 mM Tris base, 1.92 M glycine, 20% methanol, pH 8.3) at 100 V, 4 °C for 1 h. The membrane was washed twice in MilliQ water, then blocked for 2 h with protein-free blocking buffer (Quantum Scientific, Murarrie, Queensland, Australia) at AT. Following three rinses with TBST, the membrane was incubated with rabbit anti-human AGE polyclonal antibody, goat anti-rabbit IgG (diluted 1: 40,000 in protein-free blocking buffer) and substrate as in dot-immunobinding assay. Bands were visualized and analyzed as described above. The membranes were stained with Ponceau S, a general protein staining dye, after western blotting to visualize the major proteins in tears.

### Western-blot detection of AGE modified proteins in 2D-SDS gels

Since the protein amount in a single sample was not enough to run a 2D gel, tears from age- and gender-matched subjects in each group were pooled into three samples to obtain sufficient quantities of each sample for 2D gel analysis. Total protein concentration of the pooled samples was detected using the Lavapep protein quantitation kit according to the manufacturer’s instruction.

Two gels were run simultaneously for each pooled sample, one (loaded with 60 μg of tear protein) for western blotting detection of AGE modified proteins and another (loaded with 250 μg of tear protein) for Sypro Ruby staining of all tear proteins. Tear samples were mixed with 80 μl and 120 μl IEF sample buffer (MiPrep M^TM^ ; Minomic Pty Ltd, Sydney, Australia) respectively. An 11 cm IPG strip pH 4–7 (Readystrips; Bio-Rad) was rehydrated in 200 μl MiPrep M^TM^ for 5–6 h. Then cup loading of all samples was performed using two cups, which were placed at the cathode and anode ends of the strip, each containing half the amount of the sample. IEF was run at 100 μA/strip at 24 °C using an IPGphore (GE Healthcare, Uppsala, Sweden) until a total Vh of at least 50,000 was reached. Strips were reduced for 15 min in 6 M urea, 2% SDS, 0.375 M Tris, 50% glycerol, 100 mM DTT, pH 8.8 and then alkylated for 15 min in 6 M urea, 2% SDS, 0.375 M Tris, 50% glycerol, 2.5% acrylamide, pH 8.8 at AT. The second dimension was run using Bio-Rad Criterion 8%–16% gels at 4 °C, 200 V for 1 h. Five μl of precision Plus Protein^TM^ unstained molecular weight markers (Bio-Rad) were loaded in every gel at the cathode end. For all tear protein staining, the gels (250 μg total protein) were fixed in 7% acetic acid and 10% ethanol, stained overnight in Sypro Ruby (Bio-Rad) and then destained in 7% acetic acid and 10% ethanol before imaging using a proXpress CCD imager (excitation 480 nm, emission 620 nm; PerkinElmer Life and Analytical Sciences, Boston, MA). For specific western blotting detection, proteins from the gel (60 μg total protein) were transferred to NC membrane and then AGE modified proteins detected using anti-AGE antibody as described above.

### Blot image analysis

Protein spot analysis on NC membranes was performed using Progenesis SameSpots 2.0 software (Nonlinear Dynamics Limited, Newcastle upon Tyne, UK). The process included SameSpots detection, background subtraction, normalization and matching. Matching of the spots was performed using automated image alignment with manual adjustment. Differences in levels of protein expression between the three groups were analyzed by one-way ANOVA. Differences were considered significant at p<0.05.

## Results

### Dot-immunobinding assay of total AGE modified proteins in tears

**Figure 1:**
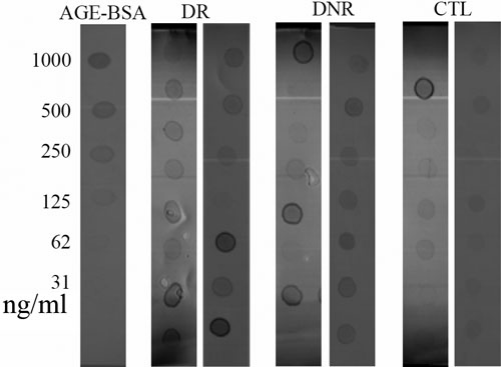
Images of dot-immunobinding assay of total AGE modified proteins in tears from DR, DNR, and CTL subjects. One dot represented a tear sample from a subject. The left lane was the AGE-BSA standard in various amounts.

Fifteen tear samples from each group were analyzed by dot-immunobinding assay using anti-AGE antibody. The images of tears from CTL, DNR and DR patients are shown in Figure 1. Based on the dot intensity of the standard AGE modified BSA, the amount of total AGE modified proteins in each tear sample was calculated (Table 2). Compared with the CTL group, tear concentrations of AGE modified proteins were elevated in DR and NDR groups. Concentrations of AGE modified proteins were higher in DR group than DNR group but the difference did not achieve statistical significance (p>0.05). After converting the results to μg AGE modified proteins per mg tear protein, the two DM groups still had higher amount of AGE modified proteins but the statistical difference between CTL and DNR became insignificant, whereas CTL versus DR remained significantly different.

**Table 2:** Quantification of AGE modified proteins in tears.

** **	**AGE modified proteins**	
**Group**	**µg/ml tears**	**µg/mg tear protein**
CTL	3.28±1.96*	0.36±0.21**
DR	7.68±3.31	0.68±0.44
DNR	5.80±2.80	0.45±0.25

The asterisk indicates a p<0.05 versus DR and DNR, and the double asterisk indicates a p<0.05 versus DR.

### Correlation of total AGE modified proteins and clinical parameters

**Figure 2:**
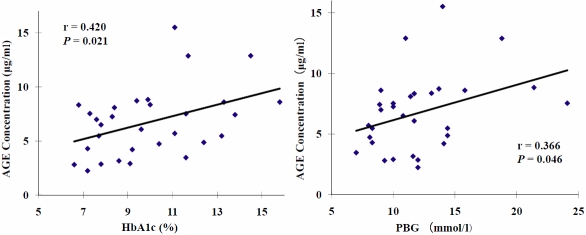
Correlation of tear total AGE modified proteins and HbA1c and PBG in DM patients with or without retinopathy.

Simple liner regression analysis showed that in these patient subgroups, the concentration (μg/ml) of AGE modified proteins in tears were positively correlated with HbA1c (r=0.420, p=0.021) and PBG (r=0.366, p=0.046) in diabetic patients (Figure 2), but no association was found between AGE modified protein concentration and FBG. However no correlation was found with any of the clinical parameters after converting the results to µg/mg tear proteins.

### Western-blotting analysis of AGE modified proteins in 1D-SDS gels

**Figure 3:**
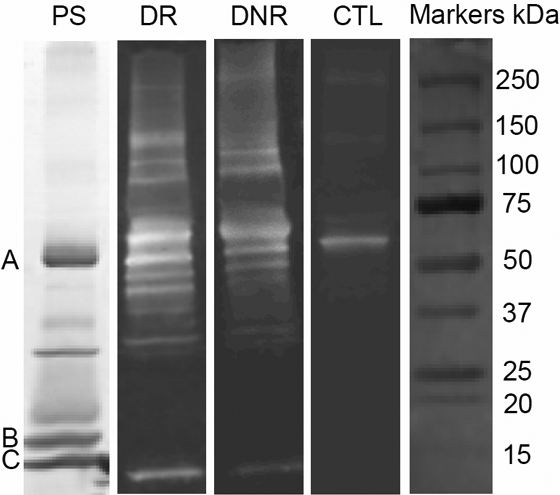
1D western blotting images of AGE modified tear proteins from DR, DNR, and CTL samples. PS: a Ponceau S stained tear proteins on the blot. A: lactoferrin, B: tear lipocalin, C: lysozyme.

A further set of tear samples, 3–5 in each group, was separated in 1D-SDS gels and AGE modified proteins were detected using anti-AGE antibody after transferring to NC membranes. Representative images of the blotting of three tear samples, one from each of the groups, are shown in Figure 3. Consistent with the dot immunobinding assay, more and stronger AGE modified protein bands could be seen in the tear samples of DM with or without retinopathy than CTL samples, indicating more proteins were AGE modified and the modification was higher in DM tears. A major band with a molecular weight (MW) of around 60 kDa appeared in every gel. A weak band approximately at the position of 13 kDa was also visible in DM tears. Ponceau S staining of the membrane detected three major tear proteins that probably corresponded to lactoferrin (A), tear lipocalin (B) and lysozyme (C) [38]. Similar intensities of the major tear protein bands stained by Ponceau S in each of the samples (image not shown) indicated that the higher amount of AGE modified proteins in DM tears was not due to larger amount of total proteins in the samples. No AGE-stained bands in the blot corresponded to probably location of tear lipocalin, indicating that this major protein was not AGE modified.

A NDR tear sample was used to do this western blotting detection without anti-AGE antibody. No band was detected in the membrane, indicating that the positive staining was not due to unspecific binding of the second antibody (goat anti-rabbit IgG) to some proteins in tears (image not shown).

### Western-blotting analysis of AGE modified proteins in 2D-SDS gels

**Figure 4:**
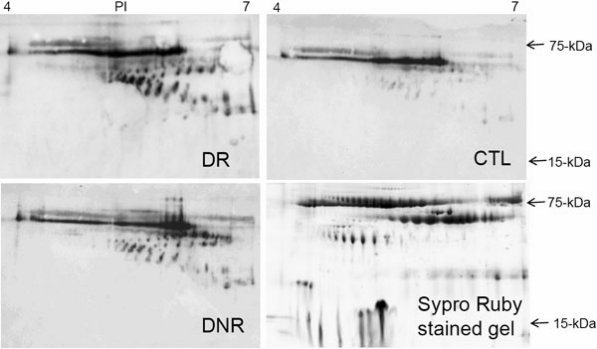
2D western blotting images of AGE modified tear proteins from DR, DNR, and CTL samples and a Sypro ruby stained 2D gel image of a tear sample.

The western blot of the 2D gels showed many spots in every sample, indicating that many protein species were potentially AGE modified, even in CTL tears (Figure 4). Compared to CTL tears, DM tears (DR and DNR) again were demonstrated to contain more AGE modified protein species. The locations of the AGE modified protein spots were very different from the location of tear protein spots in 2D gels stained with Sypro Ruby, indicating that most of the glycated proteins were of low abundance and not detectable by Sypro Ruby staining. A long strongly stained horizontal strip, which corresponded to the major band in 1D gels, at MW of approximately 60 kDa and PI of 4.5–6.2 appeared in all samples. Most of the other AGE modified protein species were within a range of molecular weight 30–60 kDa, PI 5.2–7.0 (Figure 4). The patterns of AGE modified protein staining were similar in all the tear samples but varied in intensity.

**Figure 5:**
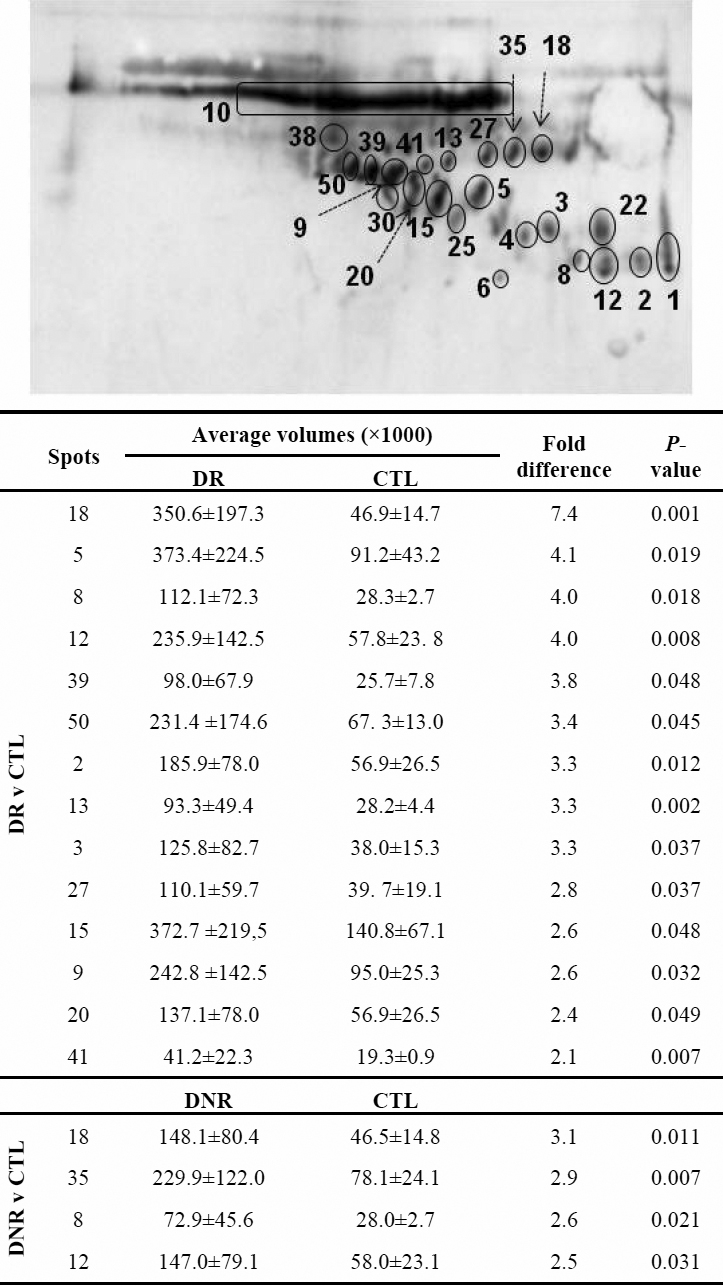
Image analysis to detect expression differences of the individual AGE modified protein spot between the groups. Only the spots showing significant differences were included in the figure table. No significant differences were detected between DNR and DR groups.

Image analysis showed that many spots were upregulated in DR tears or DNR tears when compared with CTL tears (Figure 5). This was most obvious for spot 18. Taking CTL tears as a reference, its intensity increased 7.4 fold and 3.1 fold in DR and DNR tears, respectively. The intensity of spot 5 increased 4.1 fold in DR tears but did not increase in DNR tears. Conversely, the intensity of spot 35 was elevated 2.9 fold in DNR tears but did not change in DR tears. No significant differences were detected for spot intensities between DNR group and DR group for any of the spots.

## Discussion

The results of the current study demonstrated that AGE modified proteins in DM tears are elevated in terms of the number of AGE modified protein species and the degree of modification for individual proteins. Glycation (non-enzymatic glycosylation) is a purely chemical reaction [20]. All proteins with free amino groups are subject to glycation and high tear sugar concentrations as found in the tears of diabetic subjects [18,19] would be expected to result in increased AGE modified proteins. However, the protein glycation process is slow [39] and tear turnover rate is fast [40]. There is little known about the synthesis, storage and secretion of tear proteins so information is absent about how long tear proteins already exist in the body before they are secreted into tear fluid. Some of the proteins in tears are derived from blood [7], especially in the condition of diabetic retinopathy [34,41], or released from ocular surface epithelial cells [42]. It is likely that some proteins were glycated before being secreted into tear fluid.

There were weak correlations between tear AGE modified proteins and blood HbAc1 level or PBG. Previous studies have reported that although tear glucose levels were clearly increased in DM patients, the glucose levels in blood and in tears did not always correlate [43]. This may be one reason for the weak correlation.

There are AGE modified proteins in CTL tears, although the amount was lower than in DM tears. As sugar exists in normal tears [43] and glycation is a spontaneous chemical reaction, the result is perhaps understandable. It is also well known that increased glycation is part of the aging process [44]. All the subjects in this study were elderly people and this may be another reason that AGE modified proteins exist to some extent in all their tears.

According to the spot density in the western blotting membrane, the levels of some of the AGE modified proteins change significantly between the groups, such as spot 18 (Figure 5) among the three groups, spot 5 in DR comparing with DNR or CTL. Identification of these spots by liquid chromatography combined with mass spectrometry (LC-MS) is underway and their potential as biomarkers to diagnose diabetes and/or diabetic retinopathy warrantee further studies.

Studies have shown that AGE products are themselves bioactive [45,46]. Specific receptors have been found for AGE products [47,48] and the AGE modified proteins may affect the ocular surface directly. It is also unknown whether or not the modification of tear proteins, or the present of glycation product, affects tear film stability.
